# West Nile Virus, Guadeloupe

**DOI:** 10.3201/eid1004.030465

**Published:** 2004-04

**Authors:** René Quirin, Michel Salas, Stéphan Zientara, Hervé Zeller, Jacques Labie, Séverine Murri, Thierry Lefrançois, Martial Petitclerc, Dominique Martinez

**Affiliations:** * Centre de Coopération Internationale en Recherche Agronomique pour le Développement, Petit Bourg, Guadeloupe, French West Indies; † Direction des Services Vétérinaires de Guadeloupe, Basse Terre, Guadeloupe, French West Indies; ‡ Agence Française de Sécurité Sanitaire des Aliments, Maison Alfort, France; and §Institut Pasteur, Lyon, France

**Keywords:** West Nile virus, Guadeloupe, horse, birds, serology, incidence, epidemiology, public health

## Abstract

To determine whether West Nile virus (WNV) had reached the archipelago of Guadeloupe, a serologic study in horses and birds was conducted in 2002. Immunoglobulin (Ig) G, IgM, enzyme-linked immunosorbent assay, and seroneutralization tests identified WNV infection in horses and chickens. Six months later, a high rate of seroconversion was observed in horses.

West Nile virus (WNV) was first detected on the American continent during an encephalitis outbreak in birds in New York City in September 1999 (*1*). Since then, analysis of surveillance data from 2000 to 2002 chronicles the spread of the infection to the South and the West. Infection spread to Florida, Louisiana, and the Cayman Islands in 2001 (*2*,*3*) and to northern Mexico in 2002 (*4*). Resident birds tested positive for WNV in Jamaica in 2002 (*5*), but the infection has not yet been observed in Lesser Antilles. This division of the French Indies is on the migratory route of wild birds (*6*), which are the most common vehicles for transmitting the virus over long distances (*7*). Therefore, a study with the objective of detecting the early appearance of the infection was planned in Guadeloupe on susceptible species (birds and horses) during the summer of 2002.

## The Study

A passive surveillance system of encephalitis in equine and avian species was set up to detect any occurrence of clinical signs of WNV infection. At the same time, a serologic investigation for WNV was conducted in Guadeloupe archipelago. A cross-sectional study was performed on the most susceptible animal species (birds and horses).

The survey on birds was performed in July 2002 on St. Martin/St. Maarten Island (63°5′-18°5′) on domestic ducks (Family: *Anatidae*, *Anas* species), domestic geese (Family: *Anatidae, Anser* sp.), and on laughing gulls (*Larus atricilla*), a resident wild species. The French part of the island belongs to Guadeloupe’s archipelago and is located 270 km north of the main island. It is a major resting place for migratory birds, which spend some days or weeks on the ponds before migrating south in the fall (or north in the spring). Therefore, St. Martin was chosen to increase the probability of detecting the earliest serologic conversions on resident birds and to prove the circulation of WNV among resident birds and domestic avian species. A total of 50 ducks and geese from four backyards as well as ducks from the St. Maarten Zoological and Botanical Park were sampled. On a pond, three gulls were caught and released after blood collection.

On Guadeloupe island (61°30′-16°15′), blood samples were drawn from 20 chickens from two different farms neighboring a horse-riding center in December 2002. The survey on horses was planned to be as exhaustive as possible in Guadeloupe and the nearby island of Marie Galante (61°15′-15°55′). Serum samples from 360 of 400 horses thought to live in Guadeloupe were collected in July 2002 (Figure). In locations where positive horses were detected during the first survey, another sampling was drawn from horses from December 2002 to January 2003 to measure the rate of serologic conversion and the incidence of the infection.

**Figure:**
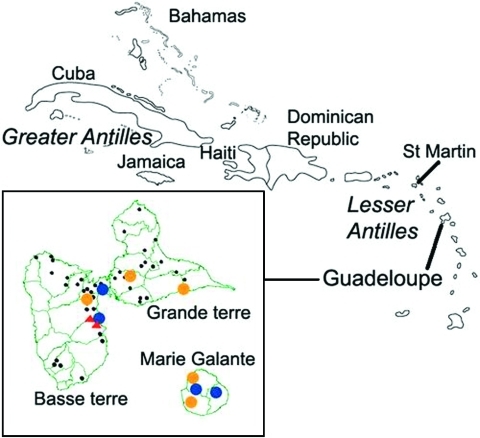
Location of Guadeloupe archipelago within the Caribbean. Insert: locations of horse and chicken sampling places in Guadeloupe and Marie Galante Islands. Red triangle, farm with seropositive chicken; blue circle, equine center with seropositive horses identified from July 2002; gold circle, equine center with seropositive horses identified from December 2002 to January 2003; black circle, equine center with no seropositive horses.

Enzyme-linked immunosorbent assays (ELISA) were performed to detect specific immunoglobulin (Ig) G antibodies to WNV in horses, ducks, geese, and chickens. Additional immunocapture IgM ELISA was performed on horses positive for WNV by IgG ELISA (*8*). Most positive serum samples were tested by plaque reduction neutralization test (PRNT 80) for both WNV and St. Louis encephalitis virus (SLEV).

All the birds (36 ducks, 14 geese, and 3 gulls) sampled from five farms and one pond in St. Maarten at the end of July 2002 tested negative for WNV by IgG ELISA (the ELISA test for the gulls has not been validated). In July 2002, 10 of 360 horses tested positive for WNV by IgG ELISA, and 2 of them were also positive by IgM ELISA. Seropositive horses were located in four different places, two in Guadeloupe and two in Marie Galante.

The results of the survey undertaken in December 2002 to January 2003 in equine centers where positive animals were detected in July 2002 indicated a high rate of WNV seroconversion in horses in these locations (Table 1). In July 2002, the overall WNV prevalence rate (IgG ELISA) was 2.8%, reaching 10.4% in places where infected horses were found (locations A, B, C, D). In January 2003, in these and related places (where some horses were moved from the former areas in July 2002), the prevalence rate increased to 50%. On paired samples, 54 of 114 horses that tested negative in July 2002 were positive in January 2003. This finding represents a seroconversion of 47.4% within 6 months. The incidence rate calculated for the places where outbreaks were noticed (A to I) is 7.9% per month. In December 2002 and January 2003, no IgM antibodies were detected on horses positive by IgG ELISA.

**Table 1:** West Nile virus IgG^a^ antibody prevalence in horses in seven equine centers, Guadeloupe and Marie Galante, July 2002–January 2003

Place	No. tested July 2002	Positive	Prevalence %	No. tested Jan 2003	Positive	Prevalence %	Seroconversion rate %
A	25	1	4.0	23	14	60.9	59.1
B	51	2	3.9	44	27	61.4	59.5
C	7	2	28.6	7	5	71.4	60.0
D	13	5	38.5	12	7	58.3	28.5
E				10^b^	2	20.0	
F				2^b^	2	100.0	
G	4	0	0.0	5	3	60.0	60.0
H	7	0	0.0	9	3	33.3	33.3
I	6	0	0.0	24	5	20.8	20.8
Total	113	10	8.8	136	68	50.0	

^a^IgG, immunoglobulin G.      ^b^Imported after July 2002.

Chickens were collected from two backyards (10 chickens in each place) neighboring one horse-riding club where positive animals were detected. Eleven of these chickens tested positive by IgG ELISA in December 2002.

To confirm the specificity of the results, positive horses samples were tested by PRNT against WNV and SLEV; all the animals showed a higher titer for WNV than for SLEV (Table 2). Specimens from 7 of 10 horses were considered positive for WNV by ELISA. Four chicken serum samples were tested by seroneutralization and confirmed positive for WNV.

**Table 2:** Results of neutralization tests for antibody to WNV and SLEV in serum samples from chickens and horses, Guadeloupe, 2002^a^

Species	Titer to WNV	Titer to SLEV	Interpretation
Chicken	>640	<20	WNV
Chicken	320	<60	WNV
Chicken	160	<40	WNV
Chicken	>640	20	WNV
Horse	160	<20	WNV
Horse	>640	<20	WNV
Horse	40	<20	Flavivirus
Horse	320	<20	WNV
Horse	320	<20	WNV
Horse	40	<20	Flavivirus
Horse	160	<20	WNV
Horse	160	<20	WNV
Horse	<20	<20	Negative
Horse	80	20	WNV

^a^WNV, West Nile virus; SLEV, St. Louis encephalitis virus.

## Conclusions

The serologic survey conducted on horses indicated an active focus of WNV infection in Guadeloupe, probably linked to the first infestation of the archipelago by the virus. The absence of IgM antibodies in horses at the end of 2002 indicates that the seroconversions did not occur during the last weeks of the year but earlier. These results (i.e., the presence of IgM antibodies in 2 of 10 positive animals in July 2002) suggest that the first WMV infections in horses probably occurred during the first 6 months of 2002 and spread in the equine population in the middle of the year.

When birds migrate, they cross the Lesser Antilles (*6*). A migratory bird, infected before leaving North America or the Caribbean Islands, may develop viremia when reaching St. Martin, Marie Galante, or Guadeloupe islands and transmit the virus to mosquito vectors during the resting period. Migratory birds from the North usually arrive in Guadeloupe later than July; thus, the infection observed in horses in July 2002 was probably not derived from migrating birds that year. WNV was probably introduced into Guadeloupe in the fall of 2001. After one or more introductions, the virus may have gradually spread in the local vector populations and amplified in resident birds even over the winter, when vectors are still active in the Caribbean. Then, 6 months later, the virus spread to susceptible species (horses), in which it was first detected.

In Guadeloupe, both animal and human surveillance systems have been set up and are interacting to detect virus circulation. In that respect, the surveillance of susceptible animal species can provide important indicators for the possible appearance of the disease in humans. As shown in the United States, the death of wild birds is a pertinent indicator for human risk (*9*). In avian species, mortality and sentinel surveillance has thus been set up. Abnormal death counts have not yet been observed. This could be related to the absence in Guadeloupe of species known to be extremely susceptible to the infection (Corvidae), vector competence, or the virus strain. A random survey is being implemented on domestic birds to assess the geographic distribution of the infection, in June through July 2003 (beginning of the rainy season), when populations of vectors increase markedly in Guadeloupe. A new serologic prevalence survey in horses is also in process, and clinical surveillance is ongoing. Despite a high rate of WNV-seropositive animals, no clinical disease has been observed. This situation could be related to the virus titer, the rate of infected vectors (which could be too low during the first year after WNV is introduced), or the virus strain. In 2003, mosquito surveillance was implemented in places where deaths in birds or encephalitis cases in horses were observed. Virus detection using reverse transcription–polymerase chain reaction will be used in our laboratory to test dead birds and pools of mosquitoes. These surveys are intended to provide the public health services with distribution and prevalence maps.

## References

[1] Centers for Disease Control and Prevention. Outbreak of West Nile-like viral encephalitis—New York. MMWR Morb Mortal Wkly Rep. 1999;48:845–9. 10563521

[2] Centers for Disease Control and Prevention. West Nile virus activity—eastern United States, 2001. MMWR Morb Mortal Wkly Rep. 2001;50:617–9. 11787570

[3] Centers for Disease Control and Prevention. West Nile virus activity—United States 2001. MMWR Morb Mortal Wkly Rep. 2002;51:497–501. 12079245

[4] Blitvich BJ, Salas IF, Cordero JFC, Marlenee NL, Rojas JIG, Komar N, Serologic evidence of West Nile virus infection in horses, Coahuila State, Mexico. Emerg Infect Dis. 2003;9:853–6. 12890327 10.3201/eid0907.030166 PMC3023419

[5] Dupuis AP, Marra PP, Kramer LD. Serologic evidence of West Nile virus transmission, Jamaica, West Indies. Emerg Infect Dis. 2003;9:860–3. 12890329 10.3201/eid0907.030249 PMC3023422

[6] Raffaele H, Wiley J, Garrido O, Keith A, Raffaele J. A guide to the birds of the West Indies. Princeton (NJ): Princeton University Press; 1998.

[7] Malkinson M, Banet C, Weisman Y, Pokamunski S, King R, Drouet T, Introduction of West Nile virus in the Middle East by migrating storks. Emerg Infect Dis. 2002;8:392–7. 10.3201/eid0804.010217 11971773 PMC2730252

[8] Murgue B, Murri S, Zientara S, Labie J, Durand B, Durand JP, West Nile in France in 2000: the return 38 years later. Emerg Infect Dis. 2001;7:692–6. 10.3201/eid0704.010417 11585534 PMC2631744

[9] Mostashari F, Kulldorff M, Hartman JJ, Miller JR, Kulasekera V. Dead bird clusters as an early warning system for West Nile virus activity. Emerg Infect Dis. 2003;9:641–6. 12781002 10.3201/eid0906.020794 PMC3000152

